# Retraction: COVID-19 knowledge, attitude and frequent hand hygiene practices among taxi drivers and associated factors in urban areas of Ethiopia

**DOI:** 10.1371/journal.pone.0336824

**Published:** 2025-11-17

**Authors:** The PLOS One Editors

This article [1] was identified as one of a series of submissions for which we have concerns about authorship and adherence to the journal’s first and second publication criteria.

In addition, the article [1] reports the same study and results as a previously published article [2, Expression of Concern in 3] that was not cited or discussed in [1].

In light of these issues, the *PLOS One* Editors retract this article.

All authors either did not respond directly or could not be reached.
